# The Role of the Teacher in the Implementation of a School-Based Intervention on the Physical Activity Practice of Children

**DOI:** 10.3390/ijerph17197344

**Published:** 2020-10-08

**Authors:** Tegwen Gadais, Theo Caron, Marie-Belle Ayoub, Antony Karelis, Luc Nadeau

**Affiliations:** 1 Department of Physical Activity Sciences, University of Quebec in Montreal, Montreal, QC H3C 3P8, Canada; Ayoub.marie-belle@courrier.uqam.ca (M.-B.A.); karelis.antony@uqam.ca (A.K.); 2 Faculté des Sports et de l’EP, Université d’Artois, 62800 Liévin, France; theo.caron0@gmail.com; 3 Department of Physical Education, Laval University, Quebec City, QC G1V 0A6, Canada; Luc.Nadeau@fse.ulaval.ca

**Keywords:** physical activity, team pentathlon, sedentary youth, intervention strategy, program implementation

## Abstract

Medium- or long-term intervention strategies for physical activity practice (PAP) need to be more effective in terms of their implementation by practitioners. The aim of this study was to evaluate the role of a teacher to implement the Team Pentathlon (TP) in order to improve the PAP in primary children. TP is a health education program made to improve PAP of children through individual and collective achievements. In this study, 203 children (age: 10–13 years) in grades 5 and 6 (intervention group (IG) *N* = 104, control group (CG) *N* = 99) were guided to increase their PAP during an eight-week period by five elementary school teachers (physical education or classroom) who had received four training sessions. Levels of PAP (self-reported) were compared between groups (IG/CG), sex, socioeconomic status of the schools and between teachers: baseline and during TP. Several teachers noted significant increases in PAP in the IG for both boys and girls (*p* ≤ 0.05 or *p* < 0.01), whereas others found only small improvements in PAP. One teacher even observed higher PAP in the CG. Training session records revealed that the teacher himself, how the TP is implemented, and proper resources were the three elements that explained the successful implementation of the TP program. The implementation of the TP significantly increased the PAP in primary children. Training sessions helped teachers to implement the TP program but personal engagement, motivation, respecting protocol, and an adequate environment are also necessary in improving the PAP of children.

## 1. Introduction

Sedentary behavior in youth has been well documented over the last two decades [1,2,3,4]. Indeed, children and adolescents do not meet the current physical activity recommendations by the World Health Organization [5]. Being in a sedentary state appears to be associated with youth obesity, which is considered one of the major issues of public health for the 21st century by the World Health Organization (WHO). Furthermore, youth obesity may lead to an increase risk of adult obesity late in life [6,7,8]. In addition, sedentary behavior and/or obesity in youth increases your risk of death, cardiovascular diseases or type 2 diabetes. It also has a negative impact on physical, psychological, and social dimensions [9,10,11,12], as well as an economic cost [3,13]. In order to counter these major issues, intervention strategies (IS) were developed and implemented in various sectors such as education, health, and public administration [14,15,16]. Over the last decade, many evidence based studies have determined that the implementation of these IS are associated with an increase in physical activity levels [16,17,18]. Moreover, a systemic review found that school based programs appear to be one of the most cost efficient IS in increasing physical activity levels [19]. Other studies have also found which IS seems to be the most effective in girls, young adults, or students [20,21]. However, the specific factors that could explain the successful implementation of these IS in children and adolescents are presently unclear [15,22,23].

### 1.1. Factors That Influence Intervention Strategies Implementation

Emerging evidence has identified several factors that could explain why certain IS are more effective in increasing physical activity levels than others. For example, Naylor et al. [15] identified 22 factors that may influence the implementation of school-based interventions in their review. Interestingly, the top seven factors were directly related to the teacher per se (characteristics, actions and his opportunities in the environment), making this individual a major factor in the success or failure of an intervention program. In particular, his time (teacher overload) or availability, his training, having a supportive school environment, technical support, his skills proficiency, as well as his own characteristics, level of commitment, and motivation have been found as major elements to explain the successful implementation of intervention programs [15]. Another review focused on the characteristics of teachers training in school-based education interventions to improve fundamental movement skills and physical activity practice (PAP) [24]. The authors from this review found that teacher training that provided pedagogy content, theoretical models or frameworks, follow-ups, and technical support to the teacher during the intervention programs were the most effective factors for improving the students PAP [24].

### 1.2. Team Pentathlon Background

Team Pentathlon (TP) is a program that normally lasts eight consecutive weeks during which team members (five or six students per team) must collectively accumulate at least 160 pentathlon hours (HP) [25]. Also, TP takes place in a school that has a stable environment [15]. It should be noted that this intervention program has been recommended to be implemented as an IS [26]. Previous studies have shown the effectiveness of TP to improve PAP in children (Nadeau et al., 2011), independent of their sex [27,28], their socio-economic status (SEL) [29], and the season of implementation [30]. In addition, participants have reported a high level of satisfaction and motivation during TP [25,28]. Moreover, Michaud et al. (2012) analyzed teacher effectiveness to improve PAP and only two teachers out of four that were tested showed significant changes in PAP. However, the study did not provide much information on the factors that could explain the improvements in PAP since the focus was not on the teacher’s role per se. Therefore, the factors that could explain the improvements in PAP with the TP remained uninvestigated.

### 1.3. Purposes of the Study

Giving these previous elements, the crucial importance of the teacher for the success or failure of a program could be related to the role of the teacher that sets up an intervention strategy in health education by using mixed methods in order to better understand his or her influence from quantitative and qualitative perspectives.

Consequently, this study aims (a) to examine the role of a teacher in implementing the TP in elementary students and its effect on PAP; (b) to identify potential factors that could explain the teacher’s performance in implementing the TP successfully.

## 2. Materials and Methods

This study used a quasi-experimental approach, whereby teachers from the same school were randomly assigned to either the intervention group (IG, TP program) or the control group (CG, regular school program). Primary classroom teachers (CT) and physical education teachers (PET) from the Quebec City area were recruited by email to participate in this study. To participate, the teachers were required to have approval from their school administration and to demonstrate interest from at least 2 classes from the same grade level (grades 5 or 6). All teachers and students gave their written consent to participate in this study and parental consent was also obtained for students. This study has received the approval from the Ethics Committee of Laval University (#2011-079/18-05-2011).

### 2.1. Participants

A total of five teachers and 203 students (91 girls and 112 boys) from grades 5 and 6, aged 10–13 years old, from five elementary school of the province of Quebec took part in this study during the winter and spring of 2012 (Table 1). Two schools (1 rural, 1 urban) were from a high socioeconomic level (SEL), one school was considered as a medium SEL (suburbs) and two were from low SEL (1 suburb, 1 urban). Socioeconomic status of the school was classified by indexes based on the family income of the student that attend the school. These indexes (numbered 1–10) were established by the provincial government of Quebec [31].

All CT and PET (*n* = 5) worked closely with the research team during the study. They collaborated daily by helping students to complete the PAP declaration sheet and received 4 training sessions to implement the TP. At the time of the study, Teacher #1 was a woman (30 years old) with 8 years of experience as a PET. Teacher #2 was a woman (40 years old), a PET with 15 years of experience. Teacher #3 was a man (25 years old) with 3 years of experience as a PET. Teacher #4 was a woman (40 years old) with 15 years of experience as a CT. She received the same TP training as the others PET, and no difference in the implementation was observed. Teacher #5 was a woman (40 years old) PET in two schools with 15 years of experience.

### 2.2. Instruments

#### 2.2.1. Determination of PAP

The amount of PAP undertaken by students was calculated in Pentathlon Hours (PH), the unit used in TP protocol that has been validated by previous studies [25,28]. The evolution of PAP amount showed how students evolved over the course of the IS [25]. The PH unit represents a composite unit of time where the actual duration of the activity is weighted according to the assumed intensity of the exercise. It was created by devising, in collaboration with cooperating PET teachers, correction factors that ensure some energy cost equivalence between the various activities selected by the participants for the duration of each PAP episode reported. For instance, playing soccer for 60 min represents 0.75 PH (correction factor = 0.75), jogging for 60 min represents 1.00 PH (correction factor = 1), whereas walking for 60 min represents 0.25 HP (correction factors = 0.25). These four correction factors corresponded approximately to the four intensity levels proposed by the American College of Sports Medicine [32] (0.25: light, 0–2.9 METs; 0.5: moderate, 3–5.9 METs; 0.75: vigorous, 6–8.9 METs; 1: very vigorous, >9.0 METs). As part of the TP, three categories were determined depending on the amount of PAP the students achieved individually at the end of the TP [25,33,34]. Students were considered:

- “Less active”, if they have accumulated an average of 0 to 1.9 PH per week;
- “Active”, if they have accumulated an average of 2 to 2.9 PH per week;
- “Very active”, if they have accumulated an average of 3 PH or more per week.

#### 2.2.2. Procedure

**Figure 1:**
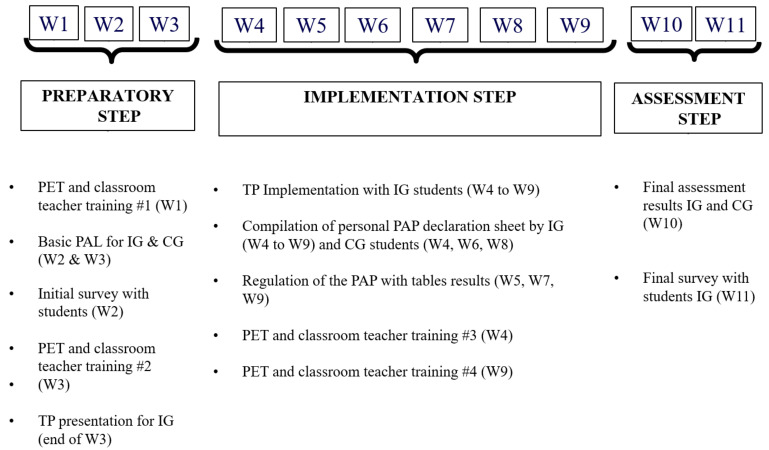
Schedule and Steps of the Study. Legends: W (Week), CG (Control Group), IG (Evaluated TP Group), PET (Physical Education Teacher), TP (Team Pentathlon), PAL (Physical Activity Level).

The study was conducted over 11 consecutive weeks and involved three steps as planned in the implementation protocol of the TP: an introduction step (3 weeks; W1, W2, W3), an implementation step (6 weeks; W4 to W9) and an assessment step (2 weeks; W10, W11) (Figure 1). Within each school, there were several groups of students that were already assigned to be in the intervention group or the control group. Therefore, the teachers were randomly assigned to the different groups of students.

#### 2.2.3. TP Training Sessions

During the preparatory step (W1 to W3), the CT and PET received training #1 to inform them about the research project (Appendix A) and collect the basic physical activity level (PAL) for participants (W2 & W3). In W3, CT and PET received training #2 to inform them about the rules for implementing the TP with PAP concepts. At the end of the same week, the CT and PET presented the TP to their students (IG) and then proceeded in creating teams, considering children motivation and peers association, that each consisted of five or six individuals. The implementation step lasted for six consecutive weeks (W4 to W9) during which each student used his personal PAP declaration sheet to report every PAP session he or she had performed the day before (Appendix C). Each morning, in the classroom, students were asked to document every session they did by reporting a) the code number of the activity and b) the duration of the period in minutes. The self-reported journals were collected by researchers every two weeks to record the PAP sessions in a computer database. This allowed the preparation of tables (Appendix B) that were provided to the students and their teacher about their participation in TP every others two weeks (W5, W7, W9). These tables periodically showed the student’s individual results and the amount of PAP by his team. The tool allowed them to compare their results to the TP standard targets [1] and, if necessary, to regulate their individual and team efforts for achieving their goals. At this stage, CT and PET received training #3 to be able to supervise regulation process with participants. At the end of the implementation step, the CT and PET also received training #4 (enabling him to conclude the TP project and administer the final TP survey). Finally, during the assessment step (W10), the teacher asked his students to make an assessment regarding their final results at TP. Students also completed a final survey about their participation in the IS. Control Group (CG) was only asked to complete the TP sheets at every two weeks during the project (W2, W4, W6, W8) without intervention or feedback from the PET.

### 2.3. Data Analysis

#### 2.3.1. Phase 1: Quantitative Data

Results are presented as means ± SD. Data analyses were conducted in two steps. For PAP participation, a frequency analysis (descriptive statistics) of PAP sessions was conducted to obtain information about the participation of students regarding their teacher and their SEL. Data were transformed to use a normal distribution (Log) and then retransformed to be presented. Independent *t*-tests were performed to compare PAP means between groups at baseline and at post values. Then, a repeated measures ANOVA was used to examine changes following the intervention within each group and between groups (time–group interaction). Bonferroni correction was applied. It should be noted that these analyses were performed separately for girls, boys, for each SEL and for the total (the two small letter notes the significant difference). Mean of W2 and W3 constituted the first measure before TP implementation (1), and W4, W6, W8 constituted the second measure during TP (2). Statistical analysis was performed using IBM SPSS Statistics for Windows, Version 22 (IBM Corp., Armonk, NY, USA). Statistical significance was set at *p* ≤ 0.05 (“a” significantly different from CG; “b” significantly different form pre values).

#### 2.3.2. Phase 2: Qualitative Data

After checking the participants PAP results that were in line with their teacher, we went deeper in the analysis by using recordings from training sessions #2, #3, and #4 that were recorded from an audio numeric system of. Training #1 was not used because it was mostly information about the research project. We transcribed the verbatim and tried to corroborate PAP results with explanations teachers gave us about the TP implementation they realised in their school. More precisely, we followed Yin (2014) recommendations to conduct a content analysis. A double blind review and coding was conducted by two authors of this study in order to identify units of sense and concepts in the transcription that could explain the teacher’s success in implementing the TP and the participants PAP results.

## 3. Results

In total, 203 students from grades 5 and 6 (10–13 years old) participated in this study. Of them, 104 (40% boys) comprised the IGs and 99 (49% girls) comprised the CGs. In total, 53% of the children were from low SEL schools, 25% from the medium SEL, and 64% from the high SEL. Low, medium, and high SEL status were 16%, 13%, and 22% in the IGs, respectively, and 17%, 12%, and 20% in the CGs, respectively.

### 3.1. Intervention Effects on PAP

As presented in Table 2, no differences in PAP means were observed between the IGs and the CGs (*p* = 0.64) at baseline between teachers (*p* = 0.076) or SEL (*p* = 0.41). There was a significant increase in PAP for the IGs (*p* ≤ 0.05) and a significant decrease in PAP for the CGs (*p* < 0.05). For CG girls, PAP significantly decreased during TP (*p* ≤ 0.05), while IG had a small improvement in PAP. IG boys significantly improved their PAP levels during TP (*p* ≤ 0.05).

### 3.2. Effects of the Teacher on PAP

For medium SEL, teacher 1 (T1) had the most effect on the PAP of children. PAP mean improved significantly for the IGs (*p* ≤ 0.05; Effect size (ES) = 0.14) and the PAP was also significant between IG and CG (*p* ≤ 0.05). Moreover, we noted that girls from the CG decreased significantly their PAP after the TP. Finally, T1 succeeded to improve significantly PAP for girls and boys from the IG (*p* ≤ 0.05). In the high SEL, teacher 2 (T2) had a global improvement of PAP but did not reach a statistical significant. We only noted significant differences between girls and boys from both the IG and CG, and a significant decrease for the CG. Teacher 3 (T3) performed slightly better with a significant difference on PAP between the IG and CG during TP (*p* ≤ 0.05, ES = 0.12). The effect could be explained mostly by a significant increase in the boys PAP level during TP (*p* ≤ 0.05). In the low SEL, teacher 4 (T4) had a global improvement of PAP confirmed by a significant difference for the IG after TP (*p* ≤ 0.05, ES = 0.09), that could be explained mostly by the significant improvement of boys PAP (*p* ≤ 0.05). Finally, teacher 5 (T5) did not succeed in improving significantly PAP and her CG had an important increase that was mostly explained by the results of boys from the CG. Others results stayed mostly stabled after TP. T1, T2, T3, and T4 improved PAP means for the IG, while the PAP of the CG declined or stayed relatively stable. Only T5 obtained a reverse tendency of the results. In this case, CG tended to improve PAP means while IG declined.

### 3.3. Factors Associated with a Successful Implementation

At this point, we analysed further the records of training sessions #2, #3 and #4 to investigate why some teachers succeeded or not to implement the TP with the participants. Content analysis allows us to identify 3 majors categories of explanations (Table 3). The first category of reasons is related to the teacher himself, namely their motivation, their level of commitment to the project, and their workload towards the TP implementation. Personal motivation seems crucial to justify the teacher’s commitment throughout the TP. For example, T1 and T3 mentioned from the beginning they were very motivated in implementing the TP. In contrast, a high amount of work or working at two schools at the same time showed a negative influence. T2 had issues to implement the project because of the schedule organisation in his school, and T5 had reported to be in a rush and almost decided to leave the project because of it.

Second, TP implementation elements are also a key in explaining the success of the program. In particular, teachers referred to five axes: (a) The implementation protocol had to be well applied (following researchers’ guidelines) and this was not always the case in this project. Some teachers decided to follow the guidelines, which resulted in good results (T1, T3, T4), whereas others rearranged the protocol, which resulted in getting negative results (T2, T5). (b) Team creation was mentioned as a reason by the teachers, but various options worked, e.g., T3 let the students make up the team, while T1 composed teams by balancing strengths and weaknesses. (c) Different strategies were used for the explanations of TP. All strategies seem to work no matter which format was presented for TP or the place to do it. For example, T1 gave TP session #1 to each separate TP teams and then explained sessions #2 and #3 to the entire group in the gym and then in the classroom. The most important was to provide complete information to students because in the case of T5, PAP episodes were not mentioned and other information was incomplete. (d) Communication between PET (in charge of TP implementation) and CT (in charge of monitoring PAP each day) is critical to ensure TP monitoring and implementation. T2 and T5 had known issues with the classroom teacher for ensuring follow-ups of the PAP information, while T1 mentioned good collaboration. T4 (classroom teacher) reported no trouble because he was the one in charge of monitoring PAP. Even more for T1, his associate classroom teacher substitute was asked by students to complete the TP sheets. Finally, (e) monitoring and follow-up was also challenging for the teacher considering the fact that they had to deal with classroom teachers on a daily basis, and monitoring has to be performed on weekends and holidays. To avoid this, teachers organised strategies like making time early in the morning to complete the sheets, noting PAP in students diary (T1, T2, T3, T4, T5), and T3 made them totally autonomous with a class routine.

Third, teachers also reported that the students themselves and facilities might influence TP implementation efficacy. Students types (not necessarily related to their SEL) could play a positive influence if they are motivated and autonomous in their follow-up in TP, or a negative influence if they don’t understand the program and what they have to do. T1 and T3 reported their students were highly motivated to participate in TP, while T2 had to frequently negotiate with students to take time in PE classes to talk about TP. Sport facilities nearby the school were also reported as an important key-element in order to make the students active. T4 mentioned many facilities were available for students such as ice hockey arena, swimming-pool or baseball field, and T5 mentioned no facilities in the nearby environment of the two schools.

## 4. Discussion

This study described the impact of an innovative school-based intervention, TP, on the PAP of 5th and 6th grade elementary school students. Results showed that students in the IG increased PAP during eight weeks of TP intervention compared to the students in the CG. We also specifically aimed to understand how a teacher’s intervention might impact the PAP of children during TP implementation.

Overall, some elements are critical to provide an effective TP implementation that could be extended to others PAP program. In a sense, this study confirmed the results of Naylor et al. [15] regarding factors that influenced implementation of school-based interventions, but also allows to go forward in knowledge development. The top seven factors found in their review such as time (e.g., competing instructional requirements, teacher overload), availability/quality of resources (e.g., activity resources, personnel, facilities), supportive school climate (e.g., shared vision/administrative support), contextual appropriateness (e.g., programme/resource acceptability), training/workshops and technical support from programme staff, self-efficacy (e.g., ease of implementation, teacher’s skill proficiency), and teacher characteristics, engagement, and motivation, were also noted in our study. In addition, the authors of this review confirmed the essential function of the teacher in charge of the program, but also the best conditions to succeed in its implementation [15,24]. Thus, the corner stone for success is a motivated teacher that is deeply convinced by the relevance of the program. Indeed, his/her commitment for the program and his/her investment with the children makes the teacher the most important factor for the success or failure of the program. Then, implementation of the program has to carefully follow the protocol steps. On one hand, PAP program creators have to carefully create implementation steps for the program and make sure they are valuable and applicable by the practitioners. On the other hand, practitioners need to respect the protocol and provide complete information. In our study, results show that the following factors need to be considered such as proper explanations, team formation, communication between actors, and the strategy to monitor and follow-up the intervention adapted to the context. For example, T1 who had the best results in improving PAP in children, was very motivated by the TP project, was deeply engaged with the children, respected strictly the protocol, took the time to explain the TP, and set up a routine with others coordinators to monitor. The environment was also favorable, which included highly motivated children and good sport facilities around the school. On the contrary, T5, who had the worst results for TP implementation (inverse tendency), was not convinced by the TP project, was poorly engaged with the children (due to the fact he was working at two different schools and was overworked), changed protocol implementation, omitted to explain some parts of TP, and tried to organize a routine, but the relationship with others colleagues was complex and students were not highly motivated. Also, T5 reported few sport infrastructures nearby the school. For others teachers (T2, T3, T4) that did a standard implementation of TP, children reached overall good results. In the present study, we showed that the context of implementation such as SEL, public or private school, urban or rural school, but also teaching experience, sex or status (i.e., PET, classroom) of the teacher, didn’t seem to affect the results of PAP in children during TP implementation, confirming the results of previous studies on TP [29,30].

Are there other potential factors that could explain the successful implementation of IS? TP is an eight-week intensive program. Thus, this timeframe could be sufficient for the children to experience obstacles, which may result in behavioral changes. This process could be important in the development of self-efficacy or intrinsic motivation for the adoption of healthy lifestyle habits [35,36]. However, children need to receive constant support from a mentor in order to preserve their commitment [15,37,38]. Second, TP includes a formal regulation process, which combines individuals and team goals, representing a core aspect of the IS. The achievement of goals can influence extrinsic and intrinsic motivation as well as the adoption of healthy lifestyle habits because meeting one’s individual goal, and contributing to the success of the team is likely to support children’s feelings of competence [37,39]. Thus, peer support, which is fostered in the design of TP, is also one of the key elements of the self-determination theory [38] and has been associated with the adoption of healthy behaviors [35]. Third, TP aims to promote the development of the students’ autonomy relating to their PAP choices. The development of autonomy is made by promoting decision making and control over behavior [37,38]. TP allows children to self-manage their individual and team progress in an “autonomy-supportive” manner [37]. Collectively, the teacher plays a crucial role in supporting children with their participation in IS and the improvement of their PAP.

### 4.1. Implications for Practitioners

This study proposed an innovative way to help students develop their autonomy in making healthy PAP choices with minimal curricular class time. Some lessons can be learned from this study:

(1) The teacher is a key person in the success or failure for the implementation of the program. To maximize chances of success, the teacher must be motivated and deeply committed to the implementation of an IS. He or she has to be engaging and supportive with children throughout the implementation. He also has to respect carefully the steps of implementation without omitting any information. In addition, he/she needs the support of his colleagues (e.g., giving time in classroom, collecting data daily) to help him with the implementation. Finally, overloading a teacher or impelling them to conduct the program could be a reason for failure in the implementation of a program [15,24].
(2) The intervention strategy team needs to design a clear and realistic implementation protocol that fits inside the school context and reality. This protocol has to be valid and strictly followed by the person in charge of the implementation [17,40]. Such an individual must respect the implementation protocol prepared by TP creators and researchers. Providing complete information to the participants with the right timing and creating good relations for collaboration tend to ensure positive results.
(3) Teachers are generally busy with their basic job. To improve the quality of implementation, certain resources are required. IS team must provide support to the teacher and help him/she with all logistical aspects of the implementation (e.g., data collection and analysis, training sessions) in order to facilitate his job [15]. In this sense, IS coordinator working outside of the school and available for the teacher, is highly recommended.
(4) Having a favorable context for the implementation is also recommended. On one hand, the school must be receptive about the IS implementation by facilitate administration tasks (e.g., authorization, communications with parents and others colleagues). On the other hand, sport facilities nearby schools can facilitate children’s PAP commitment outside of the school [15,41].

### 4.2. Strengths and Limitations

TP is mainly an extracurricular programme; thus, it takes minimal classroom time to implement and deliver the program (~1 h for each four training session over an eight-week program (six weeks in this case). This program can be implemented by the physical education teacher or by any classroom teacher who has daily contact with children. TP requires a minimum of human resources, such as a research assistant or IS coordinator to collect and enter the record sheets that are then analyzed and summarized by a computer program (~1 h/class/two weeks).

This study is not without certain limitations. First, we recruited volunteer teachers (*N* = 5) that were not randomly selected for this study. A total of eight teachers showed interest in participating at the beginning. Workload and motivation to implement TP of the teacher were not controlled in the present study, which could have influenced our results. However, we carefully addressed these issues (training session #1 and #2) with teachers before implementation of the TP. Second, teachers who had two groups of students may have been biased with the CGs and IGs because they were part of the same school. Children could have communicated together about the IS. Considering the results remained significantly different between groups suggests that either an influence did not occur or it did but did not have a significant effect.

## 5. Conclusions

This study indicated that the implementation of the TP significantly increased PAP in children. In addition, training sessions helped teachers to implement the TP program but personal engagement, motivation, respecting protocol, and an adequate environment (support from the administration and good facilities) are necessary in improving the PAP of children.

## Figures and Tables

**Table 1:** Students, Teachers and School Characteristics Involved in the Project.

School—Socio Economic Level (SEL)—Teacher	Students Groups (Grade)	Sex of Students		Total (*N* = 203)
		Girls (*n* = 91)	Boys (*n* = 112)	
School #1—Medium SEL—Teacher 1 *	Intervention Group (5th)	14	13	27 students
	Control Group (5th)	14	10	24 students
School #2—High SEL—Teacher 2 *	Intervention Group (5th)	8	15	23 students
	Control Group (5th)	8	12	20 students
School #3—High SEL—Teacher 3 *	Intervention Group (6th)	8	13	21 students
	Control Group (5th)	10	11	21 students
School #4—Low SEL—Teacher 4 *	Intervention Group (5th)	7	12	19 students
	Control Group (6th)	12	10	22 students
School #5—Low SEL—Teacher 5 *	Intervention Group (5th)	5	9	14 students
	Control Group (5th)	5	7	12 students

* More details about teachers are given upper in the text.

**Table 2:** PAP Participation Results Regarding each Teachers, CG/IG, SEL.

		Girls			Boys			Total		
	Groups	N	Mean HP Before	Mean HP During	N	Mean HP Before	Mean HP During	N	Mean HP Before	Mean HP During
Teacher 1	CG	14	3.12	2.17 ^b^	10	4.11	4.13	24	3.53	2.99
	IG	14	2.77	3.90 ^b^	13	5.27	7.35 ^b^	27	3.97	5.56 ^ab^
Teacher 2	CG	8	2.30	1.78	11	5.05	3.96 ^b^	19	3.89	2.98 ^b^
	IG	9	2.29	2.56	15	4.74	6.09	24	3.85	4.80
Teacher 3	CG	9	2.59	2.16	12	1.85	2.30	21	2.17	2.24
	IG	8	1.76	2.08	13	3.06	4.68 ^b^	21	2.59	3.77 ^a^
Teacher 4	CG	12	2.79	2.52	10	4.19	3.79	22	3.42	3.10
	IG	7	3.09	3.52	12	3.34	5.38 ^b^	19	3.25	4.70 ^b^
Teacher 5	CG	5	4.27	3.82	7	3.15	4.20	12	3.49	4.04
	IG	5	1.88	1.70	9	4.22	4.24	14	3.25	3.33
**Total**	CG	49	3.01	2.49 ^b^	50	3.67	3.68	99	3.28	2.98 ^b^
	IG	42	2.34	2.75	62	4.13	5.54 ^b^	104	3.44	4.58 ^b^
**Grand Total**	Total	91	2.68	2.62	112	3.90	4.61 ^b^	203	3.69	4.51

Legends: CG: Control Group; IG: Intervention Group—Significance between groups (*p* ≤ 0.05; “a” significantly different from CG; “b” significantly different form pre values).

**Table 3:** Teacher’s Reasons to Justify their Performance in TP Implementation.

Major Categories	Under Categories	English Translation of Verbatim
**Teacher**	*Personal motivation*	(T2) The teachers didn’t bring it up either, you know, sometimes we give ourselves challenges, but this time, there were none. (T4) The kids knew that I jog every day, and they asked me during recess if we could run together. Can we do this or …? Yes, yes, of course. It really helped the motivation.
	*Workload*	(T2) And when I used to do that, well me neither, I’m like her, I’m loaded so you know, I have no free time to go anywhere else (…) because, that’s it, me I don’t have the time to walk around all over the school. (T5) I was overwhelmed, I even wanted drop the project, I told you (…) I didn’t have any other moment because when I’m in that school, I have no free time. (…) at the other school I have a free time before dinner. (…) But the thing is I don’t have any time at this school. When I’m at this school, I have 5 periods, you know (…) no free time to go. So it had to do it in the gym.
	*Investment/engagement*	(T3) It was super easy, I was already prepared, (…) before since we saw each other the 1st time. (T4) Well it’s my fault, I should’ve left them at the secretariat. (T2) I even made myself a chart with … on top of their chart I put a cardboard with the rewards they could reach. Both the individual level and the team. Anyway, I hope it’s going to motivate them. (...) Well it’s because it’s all papers that I made for them.
**TP implementation**	*Implementation protocol*	*Conversation #2:* (R) You didn’t make any adaptations? (T1 answer) No (…) The first time, I went in class and I really followed the plan #1. *Conversation #3:* (T5) Hum no, I relied on the plan (…) Ah, in order, I did it in the order that. (R) That was presented in the plan (T5) Yes. (…) (R) Did everybody send them? (T5) No, because I didn’t do my second regulation. (…) (R) And what system, T5, did you use for the Easter period? They left with a particular system? (T5) They had to write it in their agenda. (T4) It’s quite easy, it is part of their routine. We start our day, we declare yesterday’s physical activities. It became a routine.
	*Team formation*	(T2) With friends (…) they made their teams. *Conversation #5:* (T3) The teams were formed quickly and it started. (R) Ok. So it went well for that, they are really motivated (…) (T3) Oh yeah (…) (R) So it’s the students who made them themselves? *Conversation #6:* (T5) I made them. (R) Ok. On the base of? (T5) On the base of, I put the more athletics with the less athletics. (R) Ok. You balanced the teams. *Conversation #7:* (R) Paired according to complementary team groupings? (T1) Yeah. But, you know, it’s because, there’s like one who does it all, otherwise there are 3 categories where people never do anything.
	*Explanations*	(T2) I wasn’t doing it in the gym, I had the class, but I was doing it during their outdoors period because they have like 2h20, but they were constantly whining. I even had to put my foot down. It wasn’t pleasant. (T3) On the other hand, the CM2 did that with their teacher. I went in class in class to implant it, and now they have their paper. (…) it lasted 20–25 min. They were placed by team with each their own sheet and, well, I have a good group, it’s smooth. (…) I did that in the gym. (…) Well no, because I told them to write on a paper somewhere. (…) Yeah, they left Thursday, and I told them to write Friday, Saturday, Sunday, Monday, Tuesday, when we see each other you have to write it all, you know. *Conversation#9:* (T5) but I didn’t talk about the hours, I didn’t really. (R) The HP? (T5) Yeah. (…) I explained it in 2 times (…) Well I explained in the class (…) So I went to explain it in the beginning of a class, and then when they came into class, in my class, I took over from for another 15–20 min to reexplain. (…) I had taken half an hour in one and half an hour in the other (…) Well I did the first one in the gym. It took 20–25 min. (…). Well, I still talked about it a little, but without explaining, you know, the prices that they could get. I still explained a little that it has to be done in teams, but that e didn’t have enough points.
	*Communication*	(T2) Well, me it’s because I went on vacation and I kind of left that in the hands of the teacher, but she like did not verify the … I don’t know if she was stressed or if she had something important to do. She just sent it directly to the secretariat without making the students complete them. *Conversation#11:* (T4) I think Mrs Classroom teacher didn’t give them. (R) She has to give them in pairs. (T4) Ok, so I will ask her to give them to me (…) (R) You gave me the questionnaires, is there Mrs Classroom teacher’s? These ones? (T4) No. That’s what I mean, I will have to claim them. I didn’t receive them. (…) (T4) Me, I’m missing 2 (sheets) of the witness group that might have been lost. Yes, it was the weeks when Mrs Classroom teacher was replaced, and unfortunately, I think the substitute teacher didn’t check properly or … kid name #1 and kid name #2? It’s lost.
	*Monitoring and follow-up*	(T3) Yeah, yeah. Their thing is in the morning, I pass by from time to time. *Conversation#13:* (R) You accompanied the teams, so when they were filling it up, you were there. (T1) Yeah. (T4) I obviously had the sheets, Mrs Classroom teacher had done her homework well. I made sure that all the students had handed it. And mine too (…) the way I made the feedbacks on my part was in class, of course. (…) The subject was brought back in class. And we discussed that each sport developed different things and I reminded them that the pentathlon aimed to make them try different things, that there are no sports with more values than others, that the important was to find what we liked.
**Environment**	*Students types*	(T2) Well, to be honest, since my groups are generally active, they like to move, to go to class and take the time to explain and to fill all that up (…) they were not very happy. (…) I realised I didn’t choose the right group (…) I had a turbulent group which was difficult. (…) the control group teacher is way more organised than the one who does the project. The other one you saw; her sheets are all completed; they’re all up to date. (T1) There are even students who wrote me letters stating they want to be chosen. So, there was lots of motivation from the get go. (T5) There’s a girl that went home and made herself a schedule. (…) She did it for her teammates too. So, they were running during recess. They are organised!
	*Sport facilities*	(T3) It’s pretty athletic, we move because there’s everything in the area. We have a pool, an arena, nature, everything is close. And Quebec City isn’t far so people move. (T4) However, they have a pool in the district you know (…) which is at a walking distance from school and the students are around 10 to 12 years old, usually their parents allow them to walk there. (…) There’s a pool, a rink and the Patro who is very close. Yeah, Patro Rocamadour, there’s lots of action there. (…) There’s the Marchand park, there are a lot of students who declare going skating, the park is right next to the school. So there’s the outdoor rink that allows … some of them spend all their winter there! *Conversation#15:* (R) versus T5 who is in a disadvantage environment and has doesn’t have access to such things. I don’t know if there’s a pool near you … (T5) Yes, yes, but they don’t go. (…) (R) Is the school turning in a dynamic way? (T5) Well, it’s mostly extracurricular in the school (…) Us, we don’t have pools … the pool is empty. (…) Well, it has never been open.

Legend: R (researcher), T1 (teacher#1), T2 (teacher#2), T3 (teacher#3), T4 (teacher#4), T5 (teacher#5).

**Figure A1:**
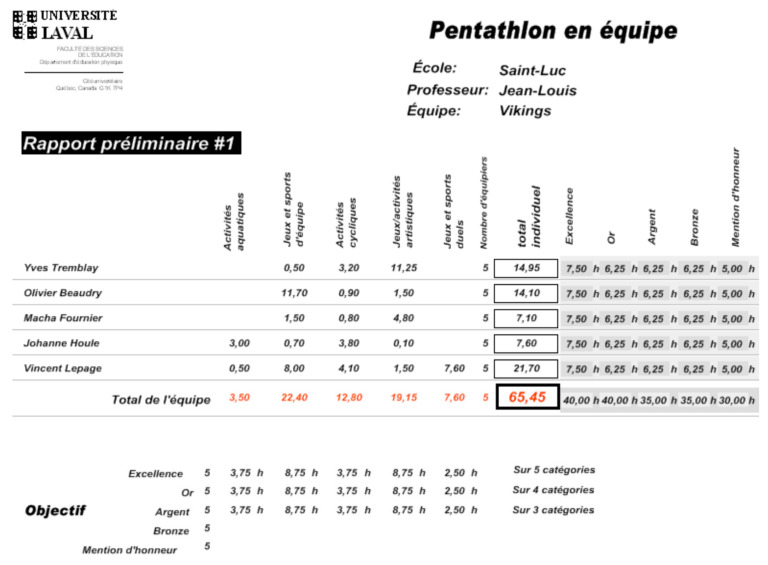
Names used in this table (student, school, teacher, team) are fictitious and are used as an example only.

## Appendix A

**Table A1:** Summary of Contents for Team Pentathlon Training Sessions.

	Contents of the Training
**Training #1** (W1)	**Project introduction** - Administrative papers (school, teacher); - General explanations about the project (3 steps); - Tasks for step #1 (Preparatory): introduce project to the students, declare with honesty about PAP, complete precisely initial survey about PAP and compile basic PAL by using PAP declaration sheet.
**Training #2** (W3)	**Team Pentathlon Implementation** *First part (tasks before beginning TP)* - Introduce TP to the students and use the student guide (objectives, individual and team goals, PAP episode, strategies, symbolic prices, correction factor); - Proceed to the composition of TP team (imposed or free to choose); - Complete the action plan sheet. *Second part (tasks during step #2 TP Implementation)* - Follow classroom teacher along step #2 about PAP declaration sheet, managing EG/CG; - Collect PAP declaration sheet along the project (after ending each steps); - Supervise PAP declaration compilation by students (every morning for W2 to W9 of the project for EG, W4, W6, W8 for CG); - Ensure that sheet and documents stay at school.
**Training #3** (W4)	**Regulation process** *First part (follow up of the project)* - Check administration paper (schools, teachers, students); - Administrate consent form (presentation process for students and parents); - Feedback about TP implementation (team composition, TP presentation with students, modification regarding the initial procedure of implementation, engagement of students, implementation contexts); - Analyze PAP declaration sheet (detect mistake or wrong information); - Establish a strategy to adjust PAP declaration compilation. *Second part (first regulation—end of W5)* - Prepare regulation process (regulation concept explanation, animation by TP responsible teacher, document stay at school); - Make assessment about PAP declaration (honesty, PH concept); - Check back the action plan they completed earlier (see if they respected it); - Talk with students about reason for not respecting their action plan and elaborate strategies to adjust it; - Explain results tables and the way to read it (group teammates together); - Explain regulation sheet #1; - Help students to complete their regulation sheet # (individual and team parts); - Encourage them to continue their good work. *Third part (second regulation—end of W7)* - Check back the action plan and the regulation sheet #1 they completed earlier (see if they respected it); - Talk with students about reason for not respecting their action plan or/and regulation #1 and elaborate strategies to adjust it; - (If necessary) Explain results tables and the way to read it (group teammates together); - Explain regulation sheet #2; - Help students to complete their regulation sheet # (individual and team parts); - Encourage them to continue their good work.
**Training #4** (W9)	**Assessment step** *First part (follow up of the project)* - Check administration paper (schools, teachers, students); - Collect all project documents (initial survey, missing PAP declaration sheet, action plan, regulation sheet #1 and #2) and prepare for the next document to come (consent form, assessment sheet, final survey) - Feedback about TP regulations #1 and #2 regarding the initial action plan. Identify reason for changing strategy and eventually not reach the target; *Second part (Assessment step—sheet)* - Assessment takes place in the classroom teacher like every meeting of TP implementation (separate the students); - (If necessary) Explain results tables and the way to read it; - Explain the assessment sheet; - Help students to complete their assessment sheet; - Acknowledge them for their good work during the project. *Third part (Assessment step—survey)* - Responsible researcher comes to complete the final survey with students (W11); - He leaves with all last missing documents and the final survey.

## Appendix C

**Table A2:** Personal Physical Activity Practice Declaration Sheet/Self-report Journal.

Team:_______________________ Name:_______________________						
**Aquatics** **(11.3 or 13.5 PH)** 1. Swimming training ^3^ 2. Swimming lesson ^3^ 3. Free swimming ^2^ 4. Synchronized swimming ^3^ 5. Inner-tube water-polo ^3^		**Team sports and games** **(26.3 or 31.5 PH)** 6. Ultimate frisbee ^3^ 7. Hockey ^3^ 8. Soccer ^3^ 9. Football ^2^ 10. Basketball-Softball ^3^ 11. Mini-volleyball ^2^ 12. Baseball ^1^ 13. Actives recess ^2^ 14. Tchoukball ^3^ 46. Kinball ^3^	**Endurance (aerobic activities)** **(11.3 or 13.5 PH)** 15. Cycling ^3^ 16. Cross-country ^4^ 17. Inline skating ^3^ 18. Free skating ^3^ 19. Speed skating 20. Mountain hiking ^3^ 21. Walking (leisure) ^1^ 22. Cross-country skiing ^3^ 23. Jogging ^4^ 47. Snowshoeing ^3^	**Individual sports and games or artistic activities** **(26.3 or 31.5 PH)** 24. Juggling ^1^ 25. Diving ^1^ 26. Rope jumping ^2^ 27. Trampoline ^2^ 28. Skateboard ^2^ 29. Gymnastics ^2^ 30. Figure skating ^3^ 31. Golf ^1^ 32. Dance ^3^ 33. Ice sliding ^1^ 34. Skiing ^1^ 35. Snowboard ^1^ 36. Hacky sack ^1^ 48. Climbing ^2^		**Dual activities** **(7.5 or 9 PH)** 37. Judo ^3^ 38. Karate ^3^ 39. Tae-kwon do ^3^ 40. Aikido ^3^ 41. Badminton ^3^ 42. Tennis or mini-tennis ^3^ 43. Ping-pong ^1^ 44. One-on-one ^1^ 45. Calisthenics ^2^
**Days of the week**	**For each physical activity session you have done, write the activity number and the duration of the session. Use one space for each different activity you perform.**					
Tuesday 27 March	No: ______ Duration: _____min, No: ______ Duration: _____min,		No: ______ Duration: _____min, No: ______ Duration: _____min,		No: ______ Duration: _____min, No: ______ Duration: _____min,	
Wednesday 28 March	No: ______ Duration: _____min, No: ______ Duration: _____min,		No: ______ Duration: _____min, No: ______ Duration: _____min,		No: ______ Duration: _____min, No: ______ Duration: _____min,	

1: correction factor 0.25; 2: correction factor 0.50; 3: correction factor 0.75; 4: correction factor 1.0.
